# Patient preference for second and third line therapies in type 2 diabetes: a prespecified secondary endpoint of the TriMaster study

**DOI:** 10.1038/s41591-022-02121-6

**Published:** 2022-12-07

**Authors:** Beverley M Shields, Catherine D Angwin, Maggie H Shepherd, Nicky Britten, Angus G Jones, Naveed Sattar, Rury Holman, Ewan R Pearson, Andrew T Hattersley

**Affiliations:** 1 Department of Clinical and Biomedical Sciences, University of Exeter, Exeter, UK; 2 Royal Devon University Healthcare NHS Foundation Trust, Exeter, UK; 3 Institute of Health Research, University of Exeter Medical School, Exeter, UK; 4 School of Cardiovascular and Metabolic Health. University of Glasgow, Glasgow, UK; 5 Diabetes Trials Unit, Radcliffe Department of Medicine, University of Oxford, Oxford, UK; 6 Population Health & Genomics, School of Medicine, University of Dundee, Dundee, UK

## Abstract

Patient preference is key for medication selection in chronic medical conditions, like type 2 diabetes, where there are many different drugs available. Patient preference balances potential efficacy with potential side effects. As both aspects of drug response can vary markedly between individuals this decision could be informed by the patient personally experiencing the alternative medications, as occurs in a crossover trial. In the TriMaster (NCT02653209, ISRCTN12039221), randomised double-blind, three-way crossover trial patients received three different second or third line once-daily type 2 diabetes glucose-lowering drugs (pioglitazone 30mg, sitagliptin 100mg, and canagliflozin 100mg). As part of a prespecified secondary endpoint we examined patients’ drug preference after they had tried all 3 drugs. 448 participants were treated with all three drugs which overall showed similar glycaemic control (HbA1c on pioglitazone 59.5 sitagliptin 59.9, canagliflozin 60.5mmol/mol, p=0.19). 115 patients (25%) preferred pioglitazone, 158 (35%) sitagliptin, 175 (38%) canagliflozin. The drug preferred by individual patients was associated with a lower HbA1c (mean 4.6 [95%CI 3.9, 5.3]mmol/mol lower vs. non-preferred) and fewer side effects (mean 0.50[0.35, 0.64] fewer side effects vs. non-preferred). Allocating therapy based on individually preferred drugs, rather than allocating all patients the overall most preferred drug (canagliflozin), would result in more patients achieving the lowest HbA1c for them (70% v 30%) and the fewest side effects (67% v 50%). When precision approaches do not predict a clear optimal therapy for an individual, allowing patients to try potential suitable medications before they choose long term therapy could be a practical alternative to optimising treatment for type 2 diabetes.

## Introduction

Patient preference is a central part of therapy selection when there are multiple suitable alternative medications for a medical condition. When expressing a preference, the patient needs to balance the likely therapeutic outcome with likely side effects. However, both therapeutic response and side effects can vary greatly between individuals so it is difficult for a patient and their physician to identify which treatment will be optimal for them. Precision medicine aims to use a person’s biomarkers or clinical features to help target the therapy they are likely to respond best to^1^, but a limitation is that patients will receive only one drug, and therefore, it is not known if they would respond better or have fewer side effects if given a different option. An alternative approach is to have a short course of the different drugs as occurs in n-of-1 or crossover trials where the patient tries multiple drugs before making a decision on which they prefer long term^2,3^. This means their decision is informed by personally experiencing the short-term benefits and side effects on each medication to help inform their treatment choice.

Treatment guidelines for type 2 diabetes emphasise the importance of taking patient choice into account^4,5^. When determining the best glucose-lowering therapy for a patient with type 2 diabetes, decisions are made balancing the glycaemic and non-glycaemic benefits of the different treatments against the potential side effects. Key factors identified by patients as important for their treatment choice include glycaemic control, weight change, risk of hypoglycaemia and gastrointestinal side effects^6,7^. A major problem when counselling patients is that both benefits (glycaemic and non-glycaemic) and side effects vary greatly between individuals and so it is not possible to give a precise prediction to the individual patient.

Information on patient preferences for specific diabetes treatments is limited. The majority of work in this area to date is based on choosing between hypothetical alternatives (discrete choice experiments or time trade off experiments), where participants are asked to rate the importance of certain attributes relating to their diabetes treatment and management^6,7^. Direct within-person comparison for determining preference of diabetes drugs, through crossover trials, has been less well studied, in part because cross-over studies are rare, with parallel group randomised controlled studies being preferred for regulatory approval. There are some examples in the literature of open-label crossover trials comparing preferences for injectable therapies^8–12^, but only one study has examined preferences of oral therapies (daily vs. once-weekly DPP4-inhibitors)^13^. There have been no randomised double-blind crossover trials to date of patient preferences for the major second and third line glucose lowering therapies (those given after metformin), which represents a key treatment dilemma in type 2 diabetes.

**Figure 1:**
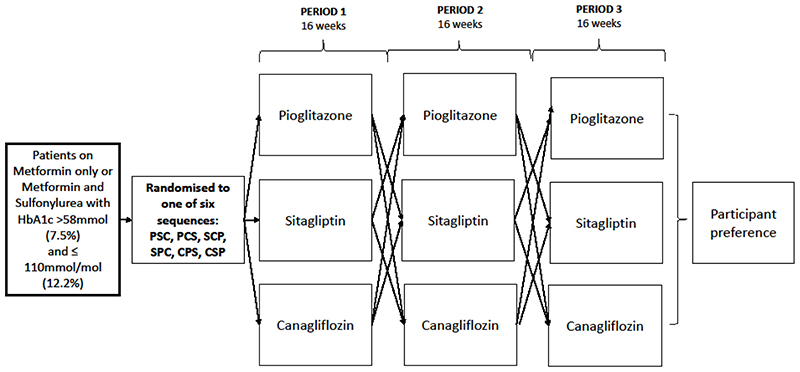
Study design for the TriMaster three-treatment, three-period crossover trial of pioglitazone, sitagliptin, and canagliflozin. Six sequences represent the 6 possible treatment orders for pioglitazone (P), canagliflozin (C) and sitagliptin (S). No washout between treatment periods (see methods). Participant preference collected at study end.

We aimed to explore patient preference for second and third line therapy in type 2 diabetes in TriMaster, a double blind, randomised three-way crossover trial of three once-daily glucose-lowering therapies: pioglitazone (30mg), sitagliptin (100mg) and canagliflozin (100mg)^14^. The primary analysis of the trial addressed clinical predictors of glycaemic therapy response and is reported separately^15^. Here, we report a pre-defined secondary analysis examining preference of participants after they had tried all three therapies in the cross-over study (Figure 1), and provide additional exploratory analysis of the associations with the patients’ preferred choice.

## Results

**Figure 2:**
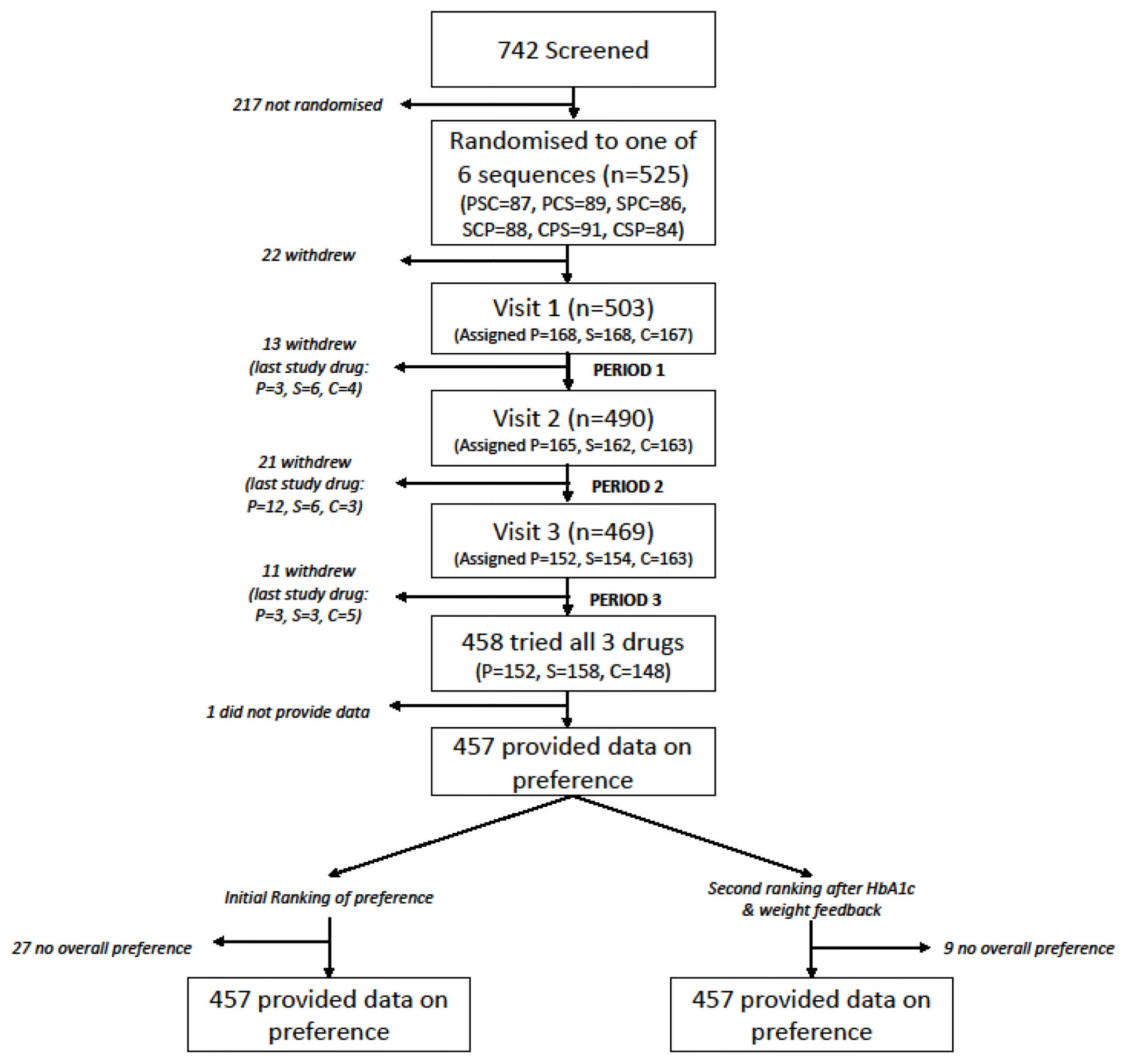
Flow diagram showing participant flow through the study, with full breakdown of numbers on each drug at each stage and exclusions. P=pioglitazone, S=sitagliptin, C=canagliflozin.

742 patients were screened between 22 November 2016 and 24 January 2020 and 525 participants randomised to the TriMaster study^15^. In total, of the 525 participants who were randomised to one of 6 drug sequences in the study, 458 (87%) tried all three study drugs, and all but one participant provided information ranking the drugs in order of preference. Figure 2 shows the flow of patients through the study. The baseline characteristics of the 457 participants who provided information on their preference are shown in Table 1. 19% would be recommended SGLT2-inhibitors in current international guidance from the American and European diabetes associations, which propose preferential choice of SGLT2 inhibitors in cardiovascular and renal disease (n=68 with atherosclerotic cardiovascular disease, n=22 with microalbuminuria/proteinuria, n=5 with both)^4^.

**Extended Data Figure 1:**
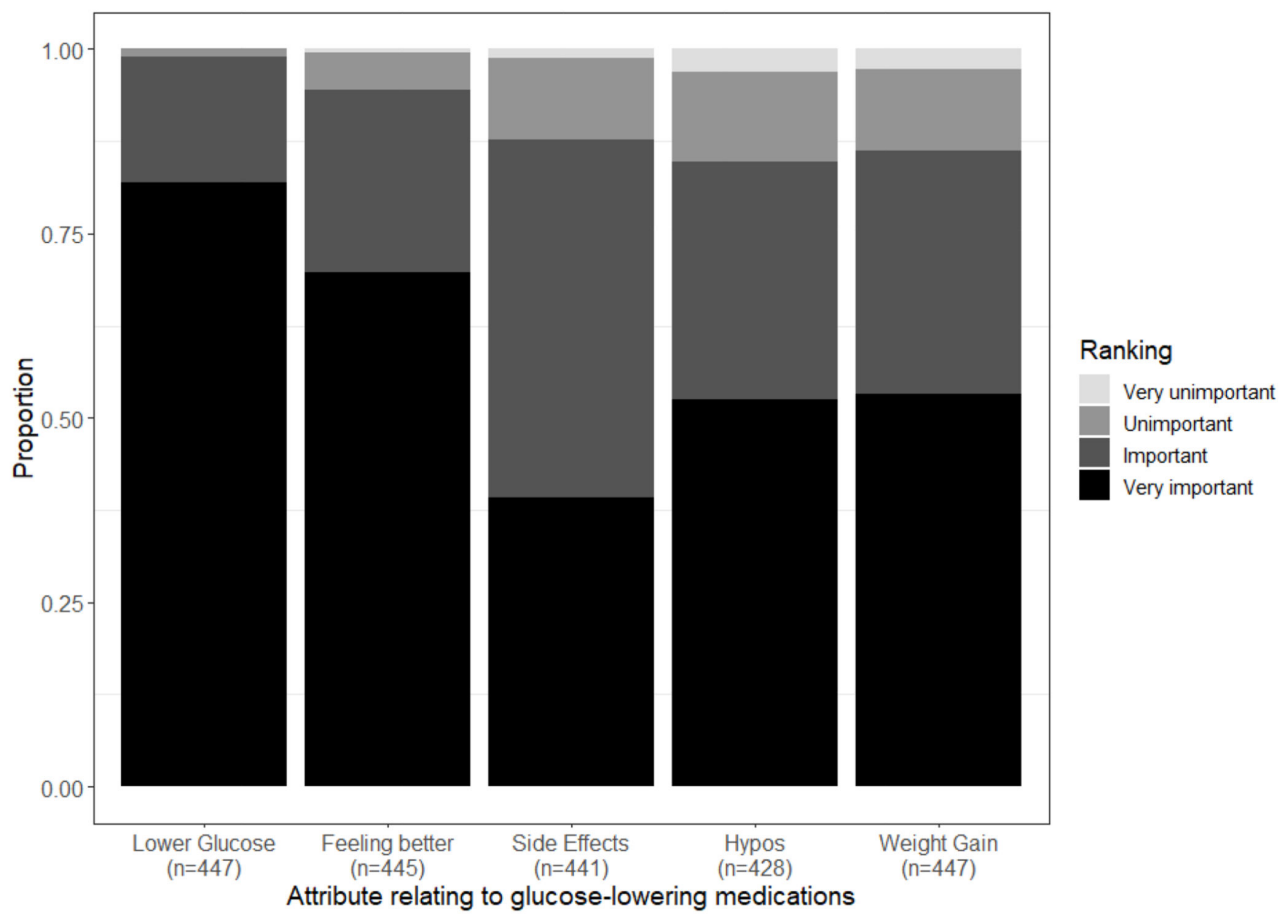
Bar chart showing, at baseline, how patients ranked the importance of 5 particular attributes when considering a glucose lowering treatment. Data presented as proportions of patients choosing each level of importance for each of the 5 attributes.

At baseline, when participants were asked their priorities for an additional glucose-lowering therapy they were most likely to rate lower blood glucose level and feeling better as very important issues with possibility of hypoglycaemia, weight gain, and side effects seen as less important (Extended Data Figure 1).

**Extended Data Figure 2:**
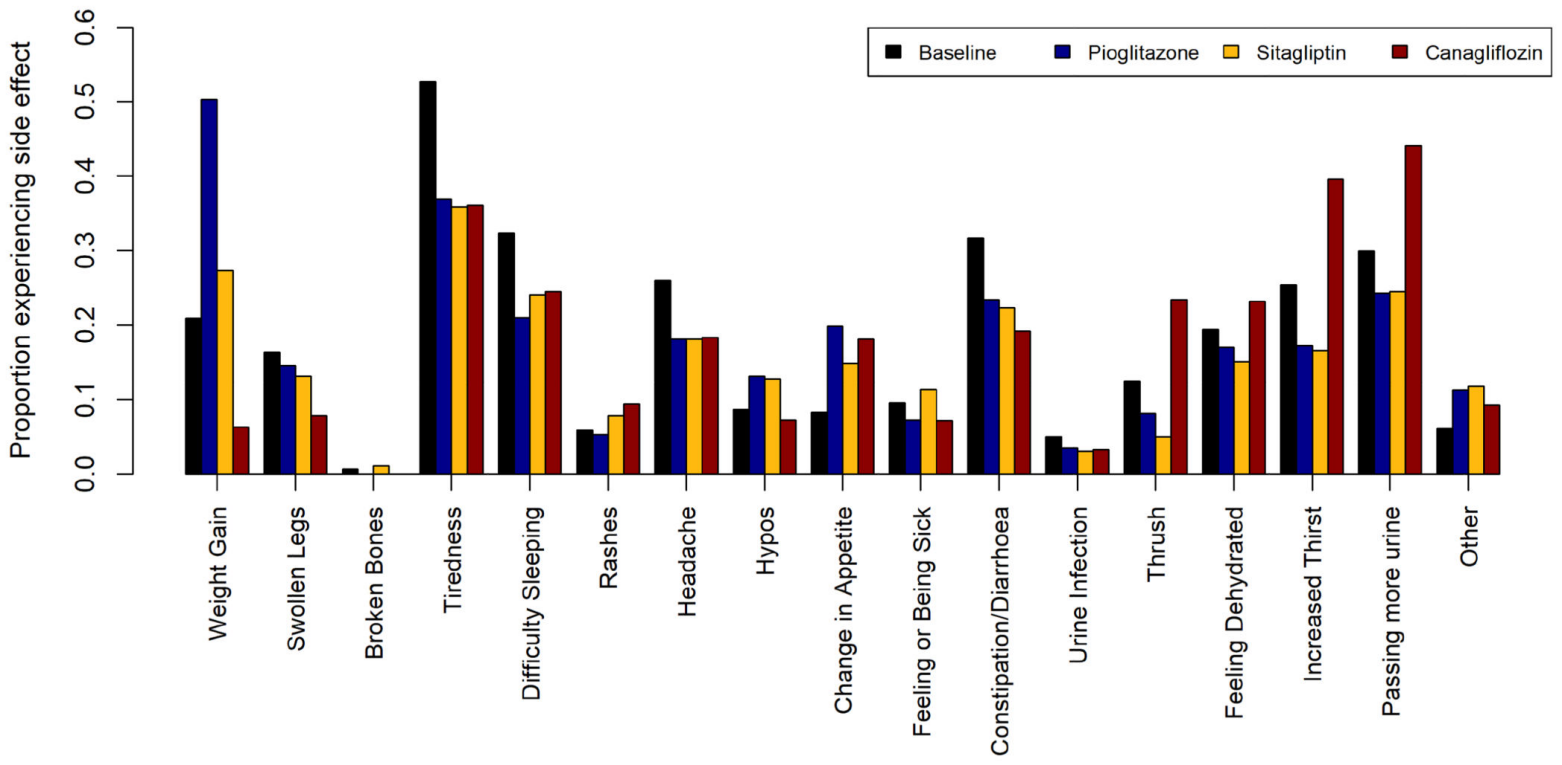
Distribution of side effects experienced on each of the three study drugs (pioglitazone represented by blue bars, sitagliptin by yellow bars, and canagliflozin by red bars) with proportions experiencing the side effects at baseline shown by black bars.

Participant characteristics recorded after receiving each of the drugs are shown in Table 1. The distribution of side effects for each drug is shown in Extended Data Figure 2. Weight gain was the most commonly reported side effect on pioglitazone. Increased thirst, passing more urine and thrush were more commonly reported for canagliflozin. Feeling or being sick was more often reported for sitagliptin.

Overall, there was equipoise between the three drugs. There was no overall difference in mean HbA1c, pioglitazone 59.6 (95% CI 58.5,60.7), sitagliptin 60.0 (95% CI 59.0,61.1), canagliflozin 60.6 (95% CI 59.7,61.6)mmol/mol (p=0.2) The lowest mean number of side effects was reported for sitagliptin. Weight was lower when treated with canagliflozin. Pioglitazone was more tolerable, with lower rates of discontinuation. When looking at within participant comparisons of the on-treatment characteristics for the three drugs, more people had their lowest HbA1c on pioglitazone, their lowest number of side effects on sitagliptin, and their lowest weight on canagliflozin (Table 2).

### Overall preference before and after being fed back HbA1c and weight

Table 3 shows the participants’ preference for the three drugs both before, and after, being fed back their HbA1cs and weights on each of the therapies. In both situations, each drug had a substantial proportion of participants who selected it as their preferred choice (range 23.6% to 38.3%). Canagliflozin was the most popular first choice, both before and after being fed back their HbA1cs and weights (37.2% and 38.3%, respectively).

There was little evidence of an order effect. 167 (37%) preferred their first drug, 148 (33%) preferred the second, 133 (30%) preferred the third (χ^2^=3.9, p=0.14 for comparison with equal preference across the three periods).

### Reasons for preference

On first ranking, 222 (52%) chose the preferred drug because of “feeling better”, whereas 164 (38%) chose it due to “lack of side effects”, with 44 (10%) unclassifiable. Canagliflozin was most likely to be chosen because of “feeling better”, whereas pioglitazone was most likely to be chosen because of “lack of side effects” (Extended Data Table 2).

After being fed back their HbA1cs and weight, 125/457 (27%) changed their preferred drug. Most patients (76%) changed because of the HbA1c result (55% HbA1c alone, 21% HbA1c and weight) while 29% changed because of weight (8% weight alone, 21% HbA1c and weight). There were clear differences between the drugs (Extended Data Table 3), with weight being most likely to result in a change in preference to canagliflozin.

### HbA1c, side-effects and weight when taking the participants preferred drug

**Figure 3:**
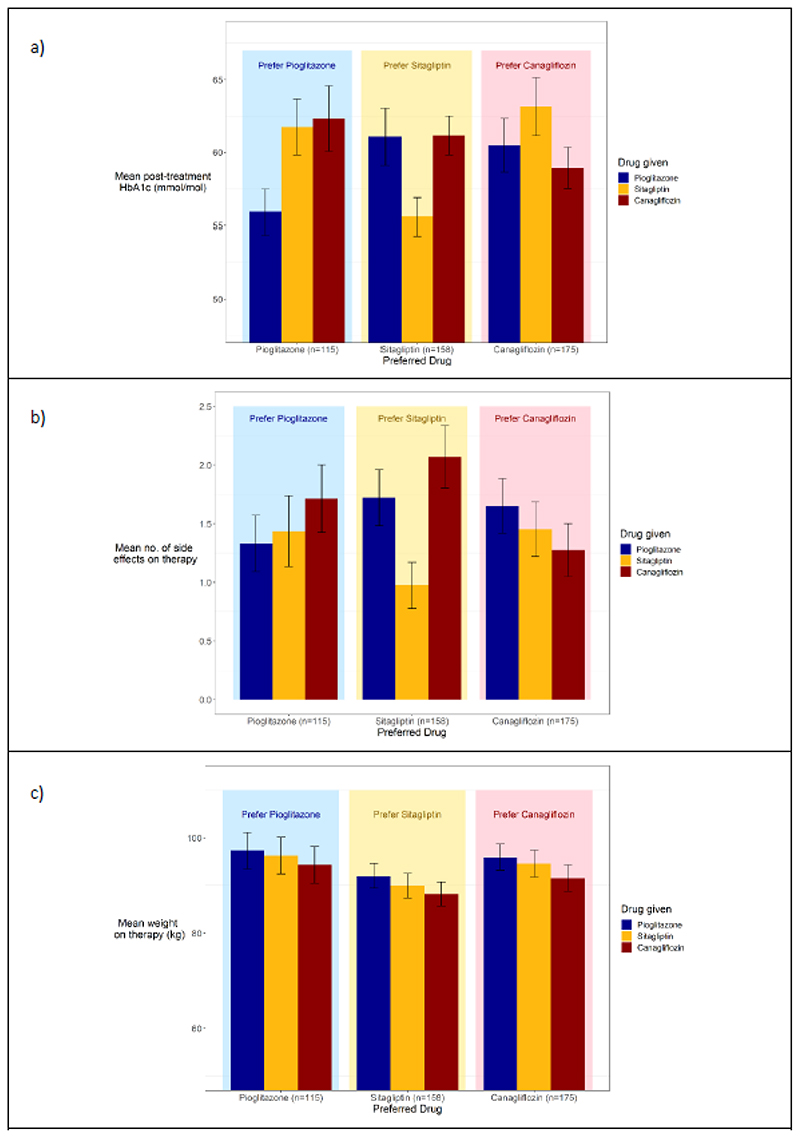
a) Mean HbA1c b) mean number of side effects and c) weight at end of treatment period for each of the three therapies (pioglitazone in blue, sitagliptin in yellow, canagliflozin in red) split by the participants’ preferred drug on final ranking (shown by light background - pioglitazone in light blue, sitagliptin light yellow, canagliflozin in pink). Error bars represent 95% confidence intervals.

There were marked differences in the glycaemic outcomes and side effect profiles in the groups of patients who preferred specific drugs (Figure 3).

**Extended Data Figure 3:**
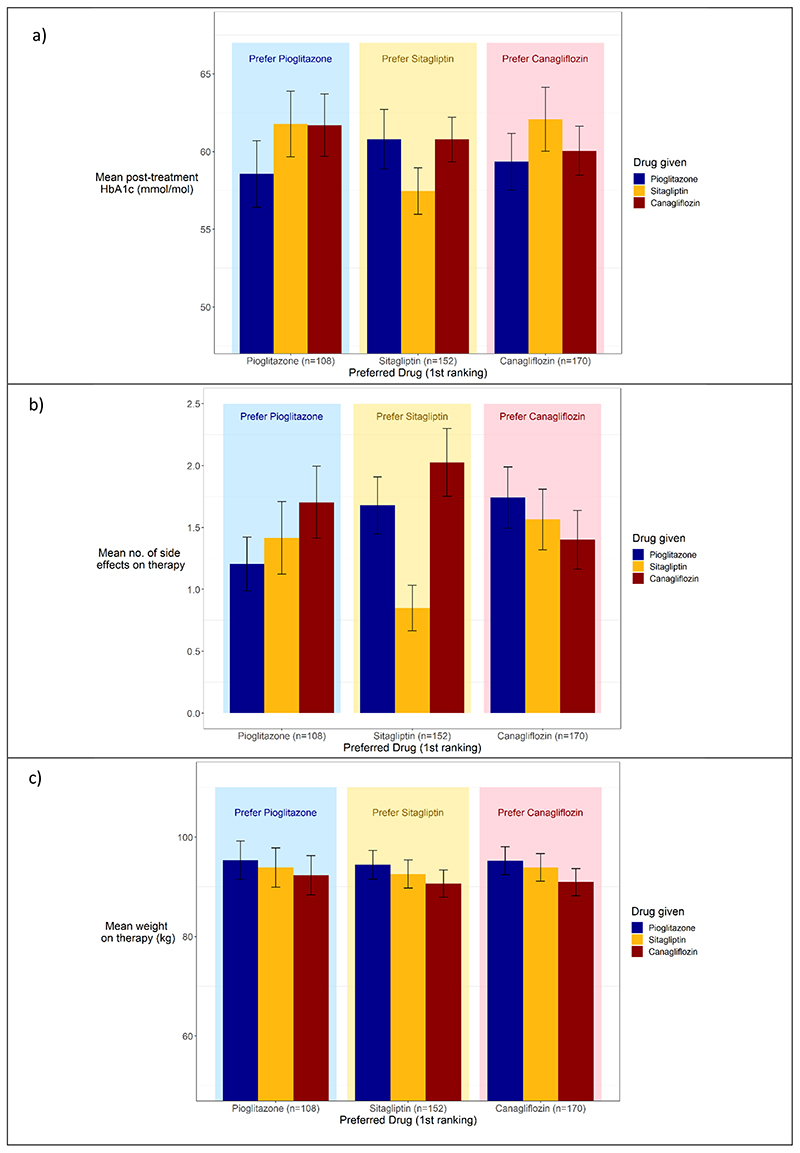
a) HbA1cs and b) Side effects on the three drugs, split by preferred therapy on 1^st^ ranking before being fed back HbA1c and weight information. Bars represent the mean and error bars represent the 95% confidence intervals.

Of the 441 participants with at least one valid HbA1c, on final ranking, after being fed back their HbA1c results, 309 (70% [95% CI 66%,74%]) preferred the drug that resulted in the lowest HbA1c for them (Table 1), and the preferred drug had a mean 4.6mmol/mol (95%CI 3.9, 5.3) lower HbA1c compared to non-preferred drugs (p<0.001, adjusting for order). The participants who preferred pioglitazone had the lowest HbA1c on pioglitazone, those who preferred sitagliptin had the lowest HbA1c on sitagliptin, and those who preferred canagliflozin had the lowest HbA1c on canagliflozin (Figure 3a). Before the HbA1c was fed back to the participants 53% preferred the drug with the lowest HbA1c, and the HbA1c on the preferred drug was a mean 2.2mmol/mol (95% CI 1.4, 3.0) lower compared to the non-preferred drugs (p<0.001). For disaggregated data by drug before being fed back HbA1c and weight, see Extended Data Figure 3a.

On final ranking, 301/448 (67% [95% Ci 63%, 72%]) chose the drug with the lowest number of side effects for them (Table 1), and the preferred drug was associated with 0.5 (95% CI 0.35, 0.64) fewer side effects (p<0.001). The participants who preferred pioglitazone had the lowest number of side effects on pioglitazone, those who preferred sitagliptin had the lowest number of side effects on sitagliptin and those who preferred canagliflozin had the lowest number of side effects on canagliflozin (Figure 3b). Before being fed back the HbA1c and weight results, 68% of those who expressed a preference chose the drug with the least side effects for them. For disaggregated data by drug before being fed back HbA1c and weight, see Extended Data figure 3b.

Weight was less of a deciding factor in patient choice, with pioglitazone associated with the highest weight on therapy, regardless of their preference (see Figure 3c). 203/448 (45% [95% CI 41%, 50%]) chose the drug on which they had the lowest weight (Table 1). The preferred drug was associated with a 1.1kg (95% CI 0.8, 1.5) lower weight compared with the non-preferred drugs when adjusting for order. Before being fed back their HbA1c and weight, 45% chose the drug with the lowest weight for them (see Extended Data Figure 4c for breakdown by drug for preference before being fed back HbA1c and weight).

## Discussion

TriMaster was a crossover study examining patient preference for second and third line glucose-lowering therapy in type 2 diabetes, enabling patients to directly compare their own lived experience of three different drugs^15^. We have shown that individual patients choose different drugs, and their preferred choice is usually associated with the drug which results in the lowest HbA1c and least side effects for them. If patients were prescribed their preferred drug, 70% would be on the drug with the lowest HbA1c and 67% on the drug with the lowest number of side effects. In contrast if all had been allocated canagliflozin (the drug most likely to be preferred in this study) only 30% would be on the on the drug with the lowest HbA1c and 50% on the drug with the lowest number of side effects. This supports a patient-centred approach to precision medicine in type 2 diabetes where a patient who has no clear clinical indication for a specific drug class would have a short-term trial of potential glucose–lowering drugs before deciding on which therapy they would prefer to take long term. This “try before you choose” approach (n-of-1) could be applicable to other chronic diseases such as hypertension^16^).

Most patients’ preference was for the drug resulting in the best glucose control. The drug with the lowest HbA1c was preferred by 53% of participants even prior to being given information on their HbA1c on the three different therapies. After being fed back HbA1c and weight, 70% preferred the drug with the lowest HbA1c. This is consistent with patients feeling better with good glucose levels and being educated that a lower HbA1c is desirable, and is in line with a recent systematic review of “discrete choice experiment” studies of patient preference in diabetes, where control of blood glucose (reflected in HbA1c) was identified as the most important attribute relating to patient preference^7^.

An unexpected result was that weight change was less important to the participants when choosing the preferred treatment. When given information on their weight on all three therapies only 45% of participants chose the drug resulting in the lowest weight for them. This is in contrast to other studies which have indicated that weight is an important attribute in treatment choice^6,7^, with 84% of patients rating weight as important in a recent meta-analysis^7^. However, these studies are based on evaluating options in hypothetical scenarios, rather than the patients comparing their own lived experience on the three drugs. At baseline in our study, prior to starting any of the drugs, the proportion of participants who rated weight as important or very important was 86%, similar to the previous studies^7^. After trying all three therapies, the patients were able to compare weight against glycaemic benefits and other side effects directly and overall, it was less important for their final preference. This outcome might reflect the relatively modest changes seen in weight in the study, potentially due to the relatively short treatment with each drug. In discrete choice studies where participants were asked to place value on glucose control compared with weight, the differences were mainly seen when considering >3kg weight loss^17,18^, whereas the weight change on the three drugs in this study was lower than this (+1.9kg for pioglitazone, +0.4kg for sitagliptin, -2kg for canagliflozin). The weight differences in our study differ slightly compared with meta-analyses of trial estimates of weight change for the three drugs (+2.6kg pioglitazone^19^, +0.55kg sitagliptin^19^, - 2.8kg canagliflozin^20^). This could reflect that the majority of trials are longer duration than 4 months.

N-of-1 trials and crossover trials have clear advantages in allowing within-person comparisons of outcomes, and have been proposed as being the ultimate way to achieve “personalised medicine” as it uses the individual’s experience of the alternative medications to inform their choice^2^ . To date, they have most commonly been used in neuropsychiatric disorders, asthma and pain management^21^. In type 2 diabetes an n-of-1 trial of potential alternative classes of glucose-lowering medication has been suggested as an optimal approach for making therapeutic decisions^3^. However, there have been no crossover preference trials in type 2 diabetes for different classes of oral medication and the approach has not featured in consensus management guidelines despite the emphasis on patient-centred medication choice^4^. The five crossover studies where patient preference was reported for type 2 diabetes treatment mainly investigated altered methods of dosing of the same therapy^8–10,12^ or alternative medications in the same class^13^. The only crossover preference study of different classes of medication was an unblinded comparison in 62 subjects offered an oral DPP4i and an injected GLP-1^11^.

N-of-1 or crossover trials are particularly attractive for studies that test precision medicine approaches that aim to target the optimal treatment for a patient based on their baseline characteristics. N-of-1 trials are appropriate for stable long-term chronic conditions, where the response to a therapy can be observed and quantified relatively quickly. The within-person comparisons they afford means they have considerable advantages over parallel group trials in terms of reduced variability and sample size. However, these trials are longer due to the sequential administration of therapies and careful consideration of study design is required to ensure sufficient washout to minimise potential carryover effects. In addition, statistical analysis can be challenging, particularly when dealing with withdrawals or missing data, as conventional imputation approaches such as ‘last one carried forward’ are not appropriate^22^.

The approach used in TriMaster^15^, allowing individuals to try several different drugs before choosing their preferred drug, could be integrated with other precision medicine strategies. Precision medicine aims to target the optimal treatment for a patient based on their baseline characteristics, and we have shown in type 2 diabetes, that simple clinical features can be used to help identify the drug likely to have the best glucose-lowering response for an individual ^23^. Using clinical features either to stratify patients or predict probability of response or side effects is an excellent approach for determining the best treatment option for an individual. However, there will still be cases where treatment decisions will be unclear as there may be little difference in predicted glucose-lowering response between different drugs; or the best choice of predicted drug for glycaemic response may carry the greatest risk of side effects (as seen with TZDs and risk of weight gain^24,25^). In these situations, where there is no clear optimal choice, the patient could choose after a brief therapeutic trial of the possible alternative medications.

Our study has not examined the long-term outcome of the try-before-you-choose approach. The next step would be a clinical trial comparing directly allocating therapy to a patient with an approach where patients can experience alternative drugs before choosing their preferred option. Further study is needed to assess if the n-of-1 approach, when applied in type 2 diabetes, results in long-term benefits that have been seen in n-of-1 studies in other diseases including increased patient empowerment, improved adherence, and reduced costs which have been seen in other diseases^26–27^.

To offer an n-of-1 type approach to patients in clinical practice would require some practical modifications to the formal clinical trial we propose. In clinical care, it would not be possible for patients to be blinded, so choice of medication could be influenced by prior beliefs of the patient or clinician regarding the benefits and risks of the therapies, but this would still allow patients to make decisions informed by their own personal experience. Given the short time periods on each drug, this approach is best for determining initial efficacy and short-term side effects. It would not be so helpful for uncommon and rare serious side-effects such as diabetic ketoacidosis, so these would need to be discussed with the patient at the time of their final preference. This n-of 1 approach will require changes in approach from healthcare professionals, but could be fitted to time with normal routine review periods (3-4 months) after initiating new therapy.

A major strength is that this is a crossover study examining patient preference for therapy in type 2 diabetes, enabling patients to directly compare their own lived experience of the three different drugs. Previous studies^6,7^ have largely used approaches where participants are asked to place value on a set of attributes based on hypothetical scenarios, so do not necessarily reflect the real-life balance of benefits and risks. A further strength is that TriMaster was a double-blind study, so patients and healthcare professionals could not be influenced by prior beliefs and information on the potential benefits and side effects of each drug.

There are limitations to our study. Since the study started, the landscape has changed with new evidence of cardiovascular and renal benefits of SGLT2-inhibitors and the cardiovascular benefits of GLP-1 inhibitors; this would be factor influencing therapy choice in patients with pre-existing cardiovascular or renal disease. However, in our study 81% of patients did not meet criteria for agents with specific cardiovascular or renal benefits, so for those at low risk of cardiovascular disease the equipoise of choice of different therapies still stands. A further limitation is that each treatment period was only 4 months, so might not have been long enough to participants to experience the full effect of the medication on glycaemic control and side effects. We only examined one drug in each class and the side effects and/or efficacy profile of other DPP4i and SGLT2i could be different. Finally, the population in our trial was predominantly (95%) self-reported White ethnicity. Further work is needed to determine whether the findings from our work are similar in other ethnicities.

In our randomised crossover trial of three oral glucose-lowering therapies in type 2 diabetes (TriMaster), we showed variability in patient preference for different drugs. Most patients preferred the drug that gave them lowest HbA1c and least side effects. In the absence of a specific indication for a particular drug, or where prediction models^28^ do not predict a clear best drug, we propose that patients could be offered a brief trial of potential alternative therapies to allow them to decide which they prefer.

## Methods

### Ethics statement

The study was approved by the UK Health Research Authority Research Ethics Committee South Central—Oxford A (16/SC/0147. The trial was registered at ClinicalTrials.gov (NCT02653209) and the ISRCTN registry (12039221).

The primary analysis of the trial, as detailed in the study protocol^14^, addressed clinical predictors of glycaemic therapy response, and is reported separately^15^. This work analyses patient preference in participants who tried all three therapies, a pre-specified secondary endpoint for the trial, and provides additional exploratory analysis of the associations with the patient’s preferred choice. The statistical analysis plan for the main trial is available for download at https://ore.exeter.ac.uk/repository/handle/10871/125162. ClinicalTrials.gov registration: NCT02653209; ISRCTN registion12039221.

### Participants

Participants were identified in primary care and from existing research cohorts in the UK. People with type 2 diabetes were eligible if aged 30-80 years on stable doses of metformin alone, or metformin and a sulfonylurea with HbA1c >58mmol/mol (>7.5%) and ≤110mmol/mol (≤12.2%). All participants gave written informed consent and were compensated for travel expenses only. Ethnicity was self-reported by participants using standard 2011 UK Office for National Statistics codes.

### Study design

Between 22 November 2016 and 24 January 2020, 742 patients were screened for eligibility with 525 randomised to one of the 6 possible treatment orders for the three therapies and asked to take each therapy for 16 weeks. HbA1c, weight, participant’s experience of the therapy, and side effects were recorded at the end of each treatment period. The 16-week treatment period was designed to allow an “on-treatment” washout for the first month, with each end-of-period HbA1c reflecting the previous 8-12 weeks on therapy. HbA1c results were only considered valid if the participant had been on the therapy for at least 12 weeks and they had at least 80% adherence, as measured by pill count at the end of each treatment period. Participants reported whether they had experienced any of a pre-defined list of 16 side effects associated with the three therapies (see Extended Data Table 4 for full list). For this study, we analysed only new side effects (i.e. those not recorded at baseline). Total number of side effects reported on each therapy was calculated for each participant. Tolerability was defined as staying on the drug for at least 12 weeks.

### Randomisation and blinding

Randomisation was carried out at the baseline visit as described in the study protocol^14^ and statistical analysis plan. The three therapies were allocated in random order according to six possible treatment orders (block size 12): ABC, ACB, BAC, BCA, CAB, CBA. Drugs were blinded by over-encapsulation (Tayside Pharmaceuticals, Dundee, UK) with allocations blinded to the participants, study team, study researchers, and study statistician.

### Sample size

Sample size was determined for the main primary analysis reported separately (submitted in parallel). This study presents exploratory analysis relating to the secondary endpoint of patient preference. Confidence intervals are reported throughout to show precision of our estimates.

### Participant preference

#### Assessment of treatment attributes at baseline

Important considerations for future glucose-lowering therapies prior to randomisation were assessed at baseline with a 1-4 Likert scale from ‘very important’, ‘important’, ‘unimportant’ to ‘very unimportant’.

Prior to each treatment period, participants were provided with a paper ‘reporting card’ to collect any noteworthy experiences or events. Completion was optional, they served as memory aide for the 16-week period. At the end of the treatment period study visit, participant experience questionnaires were completed by the participant themselves. Ahead of the final study visit, copies of documents from all three treatment periods were provided along with a summary showing whether at the time they had felt the drug had made their lives more difficult/no difference/less difficult, and whether they would be willing to take it again. Participants were asked to consider their experiences with a view to expressing a preference at the visit.

At the final study visit, participants were asked to rank each of the drugs in order of preference on a scale of 1 to 3 (1 being most preferred to 3 being least preferred). Participants were asked to rank the drugs twice: once before they received information on their HbA1c result and weight on each of the therapies and once again after being fed back these results. Main preference results are shown as both mean rank (in line with the secondary endpoint in the SAP) and preferred drug. As our main analysis aimed to explore reasons for patient preference, analysis was mainly focussed on examining associations with preference as a binary variable (preferred v not preferred drugs), as in real life patients would only continue with taking one drug. Therefore, participants that could not decide on a preferred drug were excluded from analysis. Participants were asked, for each of the study drugs, whether they would continue taking them long term.

### Reasons for preference ranking

Participants were asked for their reasons behind their ranking. Participants were still blinded to drug allocations when assigning their first ranking, so reasons were provided relating to the order they were given (i.e. their first, second or third drug). Two researchers (BS and CA) independently coded the free text responses, without information on the final drug allocations, only which of the first, second or third drugs was the preferred. Free text was coded as either: 1) “feeling better” if they stated their preferred drug was chosen for having a positive effect on their health/wellbeing; 2) “lack of side effects” if the main reason for their preference was due to the preferred drug not having side effects that the other drugs did; or 3) unclassifiable. The two sets of codes were then compared, and discrepancies resolved through discussion with a third researcher (MS).

### Statistical analysis

Summary statistics are reported as mean (for continuous variables) or frequency and percentage (for categorical variables), with 95% confidence intervals, unless otherwise stated. Characteristics (HbA1c, weight, number of side effects, tolerability) of the participants on the three drugs were compared using mixed effects models, to allow for repeated measures, with drug coded as a dummy variable and participant as the random effect. Chi-squared tests were used to compare differences in proportions against the null hypothesis of equal preference (i.e. 1/3). Mixed effects models were used to determine the difference in outcome (HbA1c, weight, number of side effects) between preferred and non-preferred drugs (binary variable, fixed effect), adjusting for study period (dummy variable) with participant as the random effect to allow for repeated measurements. All analysis was carried out using a validated version of Stata v16.1.

### Patient involvement

A TriMaster PPI group was established in 2015 from participants of earlier pilot studies and worked alongside the existing Peninsula Research Bank PPI group to assist in study design and acceptability for the trial. The group contributed to the design and content of data collected on participant experience of the study drugs, to participant information sheets and were represented on the Trial Steering Committee. Both groups reviewed study documents ahead of regulatory submission. At study conclusion a participant group was invited to feedback on their experience to the CI team.

## Extended Data

**Extended Data Table 1:** Baseline characteristics of 457 participants who provided information on their drug preference in the TriMaster trial.

Characteristic	Mean (SD), *Median (IQR), or n(%)
Male n(%)	336 (74%)
Female n(%)	121 (26%)
Age (years)	61.8 (9.5)
Age at diagnosis (years)	52.9 (8.9)
Weight (kg)	93.1 (18.2)
BMI (kg/m^2^)	31.7 (5.4)
HbA1c (mmol/mol)*	69 (63, 78)
Treatment: Metformin	221 (48%)
Metformin+Sulphonylurea	236 (52%)
Met criteria for SGLT2 treatment initiation under EASD/ADA guidelines:	
Atherosclerotic CVD	68 (14.9%)
Microalbuminuria/proteinuria	22 (4.8%)
Both	5 (1.1%)
Self-reported Ethnic Group:	
White	433 (94.8%)
Asian	12 (2.6%)
Black	2 (0.4%)
Other	6 (1.3%)
Not stated	4 (0.9%)

**Extended Data Table 2:** Reason for choosing preferred drug for 430 participants when ranking order of drugs on first ranking *before* being fed back HbA1c and weight information for all three therapies.

	Preferred Drug			
*Reason for preference*	Pioglitazone	Sitagliptin	Canagliflozin	Total
Feeling better	42 (39%)	73 (48%)	107 (63%)	222 (52%)
Lack of side effects	54 (50%)	64 (42%)	46 (27%)	164 (38%)
Unclassifiable	12 (11%)	15 (10%)	17 (10%)	44 (10%)
Total	108	152	170	430

**Extended Data Table 3:** Number changing preference by drug and reasons given for the 125 participants that changed their preference on second ranking *after* being fed back HbA1c and weight information for all three therapies

	Preferred Drug				
	Pioglitazone	Sitagliptin	Canagliflozin	No pref	Total
Preferred drug on second ranking	39	40	43	3	125
*Reason for changed preference:*					
HbA1c	30 (76.9%)	28 (70%)	11 (25.6%)		69 (55%)
Weight	1 (2.6%)	1 (2.5%)	8 (18.6%)		10 (8%)
Both HbA1c and Weight	3 (7.7%)	7 (17.5%)	16 (37.2%)		26 (21%)
Unclassifiable	5 (12.8%)	4 (10%)	8 (18.6%)	3 (100%)	20 (16%)

**Extended Data Table 4:** List of side effects patients asked to report at baseline and follow-up study visits

Swollen ankles/legs
Weight gain
Broken bones
Low blood sugar
Headache
Increased thirst
Passing more urine
Feeling dehydrated
Tiredness
Difficulty sleeping
Rashes
Change in appetite with weight change
Feeling or being sick
Constipation / diarrhoea
Urine infection
Thrush / Rash or redness of foreskin

## Figures and Tables

**Table 1:** On-treatment characteristics of 457 participants who tried all 3 drugs. Data presented as mean and 95% confidence intervals or n(%); P values determined using mixed effects models adjusting for period.

	Treatment			
	Pioglitazone n=457	Sitagliptin n=457	Canagliflozin n=457	p
HbAlc (mmol/mol)^a^	59.5 (58.4, 60.6) n=413	59.9 (58.9, 61.0) n=380	60.5 (59.5, 61.4) n=397	0.2
Discontinued therapy within 12 weeks	23/457 (5.0% [3.2, 7.5])	38/457 (8.3% [6.0, 11.2])	35/457 (7.7% [5.4, 10.5])	0.09
Weight (kg)	94.9 (93.2, 96.7) n=452	93.4 (91.7, 95.1) n=453	91.1 (89.4, 92.8) n=454	1 x 10^-183^
Number of side effects	1.59 (1.46, 1.73)	1.30 (1.16, 1.44)	1.66 (1.51, 1.81)	4 x 10^-5^
Participants stated they would take it long term:				
Yes	244 (54%)	264 (58%)	273 (60%)	0.5^b^
Maybe	125 (27%)	117 (26%)	106 (23%)	
No	83 (18%)	73 (16%)	77 (17%)	

a HbA1c only valid if on therapy for at least 12 weeks and 80% adherence based on pill count;

b P value for Yes/Maybe v No across the three drugs

**Table 2:** Number of individuals achieving specific optimal results by drug. For each drug, the number (and % [95% CI]) of participants that have the lowest HbA1c, side effects, and weight on that drug. NB participants can have the lowest value on more than one drug so row totals add up to >100%.

	Pioglitazone	Sitagliptin	Canagliflozin	Patient’s preferred drug^a^
Drug resulting in lowest HbA1c of the three drugs	203/457 44 [40-49]%	171/457 37 [33-42]%	135/457 30 [25-34]%	309/441^b^ 70 [66-74]%
Drug resulting in lowest number of side effects of the three drugs	223/457 49% [44-53]%	283/457 62 [57-66]%	230/457 50 [46-55]%	301/448 67 [63-72]%
Drug resulting in lowest weight of the three drugs	22/457 5 [3-7]%	87/457 19 [16-23]%	352/457 77 [73-81]%	203/448 45 [41-50]%

a Only presented for participants where a preferred drug was chosen.

b 7 participants had no valid HbA1cs for any of the three drugs

**Table 3:** Patient’s preference of drug before and after being fed back information on the HbA1c and weight at the end of the 3 treatment courses. Data presented as number and percentage preferred drug [95% confidence intervals] and mean rank. Ranking of drugs is 1 for preferred, 3 for least preferred, so lower mean rank represents higher preference for the drug.

	Total	Pioglitazone	Sitagliptin	Canagliflozin	No preference	P^*^
Preferred drug before HbA1c & Weight	457	108 23.6[20.0-27.8]%	152 33.3[29.1-37.7]%	170 37.2[32.9-41.7]%	27 5.9[4.1-8.5]%	0.0008
Mean rank		2.08	1.97	1.95		
Preferred drug after HbA1c & weight	457	115 25.2[21.2-29.4]%	158 34.6[30.2-39.1]%	175 38.3[33.8-42.9]%	9 2.0[0.9-3.7]%	0.002
Mean rank		2.09	1.98	1.93		

* P values calculated for those who expressed a preference, based on chi-squared test compared with equal preference.

## Data Availability

To minimize the risk of patient re-identification, de-identified individual patient-level clinical data are available under restricted access. Requests for access to anonymised individual participant data (IPD) and study documents should be made to the corresponding author and will be reviewed by the Peninsula Research Bank Steering Committee. Access to data through the Peninsula Research Bank will be granted for requests with scientifically valid questions by academic teams with the necessary skills appropriate for the research. Data that can be shared will be released with the relevant transfer agreement.

## Code availability

Requests for access to code should be made to the corresponding author and will be reviewed by the Peninsula Research Bank Steering Committee. Access to code through the Peninsula Research Bank will be granted for requests with scientifically valid questions by academic teams with the necessary skills appropriate for the research. Code will be released by the lead statistician.

## References

[1] (2011). Toward Precision Medicine: Building a Knowledge Network for Biomedical Research and a New Taxonomy of Disease.

[2] Lillie EO (2011). The n-of-1 clinical trial: the ultimate strategy for individualizing medicine?. Per Med.

[3] Tsapas A, Matthews DR (2008). N of 1 trials in diabetes: making individual therapeutic decisions. Diabetologia.

[4] Davies MJ (2022). Management of hyperglycaemia in type 2 diabetes, 2022. A consensus report by the American Diabetes Association (ADA) and the European Association for the Study of Diabetes (EASD). Diabetologia.

[5] National Institute for Health and Care Excellence (NICE) (2015). Type 2 diabetes in adults: management NICE guideline (NG28).

[6] Purnell TS, Joy S, Little E, Bridges JF, Maruthur N (2014). Patient preferences for noninsulin diabetes medications: a systematic review. Diabetes Care.

[7] Toroski M (2019). Patient and physician preferences for type 2 diabetes medications: a systematic review. J Diabetes Metab Disord.

[8] Bohannon N (2011). Comparison of a novel insulin bolus-patch with pen/syringe injection to deliver mealtime insulin for efficacy, preference, and quality of life in adults with diabetes: a randomized, crossover, multicenter study. Diabetes Technol Ther.

[9] Gentile S (2018). A randomized, open-label, comparative, crossover trial on preference, efficacy, and safety profiles of lispro insulin u-100 versus concentrated lispro insulin u-200 in patients with type 2 diabetes mellitus: a possible contribution to greater treatment adherence. Expert Opin Drug Saf.

[10] Korytkowski M, Bell D, Jacobsen C, Suwannasari R, FlexPen Study T (2003). A multicenter, randomized, open-label, comparative, two-period crossover trial of preference, efficacy, and safety profiles of a prefilled, disposable pen and conventional vial/syringe for insulin injection in patients with type 1 or 2 diabetes mellitus. Clin Ther.

[11] Ludemann J, Dutting ED, Dworak M (2015). Patient preference and tolerability of a DPP-4 inhibitor versus a GLP-1 analog in patients with type 2 diabetes mellitus inadequately controlled with metformin: a 24-week, randomized, multicenter, crossover study. Ther Adv Endocrinol Metab.

[12] Matza LS (2020). Assessing patient PREFERence between the dulaglutide pen and the semaglutide pen: A crossover study (PREFER). Diabetes Obes Metab.

[13] Meguro S, Matsui S, Itoh H (2019). Treatment preference for weekly versus daily DPP-4 inhibitors in patients with type 2 diabetes mellitus: outcomes from the TRINITY trial. Curr Med Res Opin.

[14] Angwin C (2020). TriMaster: randomised double-blind crossover study of a DPP4 inhibitor, SGLT2 inhibitor and thiazolidinedione as second-line or third-line therapy in patients with type 2 diabetes who have suboptimal glycaemic control on metformin treatment with or without a sulfonylurea-a MASTERMIND study protocol. BMJ Open.

[15] Shields BM (2022). Patient stratification for determining optimal second and third line therapy for type 2 diabetes: the TriMaster study. Nature Medicine.

[16] Samuel JP (2019). Treating Hypertension in Children With n-of-1 Trials. Pediatrics.

[17] Bogelund M (2011). Patient preferences for diabetes management among people with type 2 diabetes in Denmark - a discrete choice experiment. Curr Med Res Opin.

[18] Hauber AB, Mohamed AF, Johnson FR, Falvey H (2009). Treatment preferences and medication adherence of people with Type 2 diabetes using oral glucose-lowering agents. Diabet Med.

[19] Domecq JP (2015). Clinical review: Drugs commonly associated with weight change: a systematic review and meta-analysis. J Clin Endocrinol Metab.

[20] Zaccardi F (2016). Efficacy and safety of sodium-glucose co-transporter-2 inhibitors in type 2 diabetes mellitus: systematic review and network meta-analysis. Diabetes Obes Metab.

[21] Gabler NB, Duan N, Vohra S, Kravitz RL (2011). N-of-1 trials in the medical literature: a systematic review. Med Care.

[22] Senn S (2002). Cross-over trials in clinical research.

[23] Shields BM (2022). TriMaster: randomised double-blind crossover trial of a DPP4-inhibitor, SGLT2-inhibitor and thiazolidinedione to evaluate differential glycaemic response to therapy based on obesity and renal function. submitted in parallel to Nature Medicine.

[24] Dennis JM (2018). Evaluating associations between the benefits and risks of drug therapy in type 2 diabetes: a joint modeling approach. Clin Epidemiol.

[25] Dennis JM (2018). Sex and BMI Alter the Benefits and Risks of Sulfonylureas and Thiazolidinediones in Type 2 Diabetes: A Framework for Evaluating Stratification Using Routine Clinical and Individual Trial Data. Diabetes Care.

[26] Nikles CJ, Clavarino AM, Del Mar CB (2005). Using n-of-1 trials as a clinical tool to improve prescribing. Br J Gen Pract.

[27] Scuffham PA (2010). Using N-of-1 trials to improve patient management and save costs. J Gen Intern Med.

[28] Dennis JM (2020). Precision Medicine in Type 2 Diabetes: Using Individualized Prediction Models to Optimize Selection of Treatment. Diabetes.

